# Notes on Preparations for the Sick

**Published:** 1908-03

**Authors:** 


### Notes on Preparations for the Sick.

**Bromural.**—KNOLL & CO., London.—The drug is a derivative of bromine, valerian and the urea basis, and stands, therefore, in its action between a hypnotic on one side and a sedative on the other. Though having a slight hypnotic effect, it is absolutely harmless, and can be especially recommended in cases where a powerful hypnotic is not advisable.

Extensive experiments have been carried out lately with the drug in this country and on the continent, and the fact was established that "Bromural" can be recommended safely as an effective and harmless sedative, inducing dreamless, natural sleep without any by- or after-effects.

It acts quickly, and its action continues for several hours. The ordinary dose is one or two tablets, each containing 0.3 gm.

**Glycerophosphates** (Roberts).—ROBERTS & CO., 76 New Bond Street, London.

Syr. Glycerophosph. Co. = Syr. Roborans (Roberts).

Glycerole Glycerophosph. Co. = Glycerole Roborans (Roberts).

In the presence of so many more modern preparations of the glycerophosphates, the above-mentioned, the original preparations of Robin, should not be forgotten. The one with sugar, the other with glycerine, alike in all other respects, are excellent preparations, which have an established reputation for use in all conditions of general and nervous debility.

---

**Hémoglobine Deschiens.**—ROBERTS & CO., London.—Two preparations of this are in common use—

Deschien's Syrup, and
Deschien's Hémoglobine Wine.

Granulated Hémoglobine and Dragées are also prepared. They are all obviously useful when organic preparations of iron are desirable, as in chlorotic anæmia and neurasthenic conditions. It is a well-known fact that the inorganic preparations of iron often disagree with individual patients, and that in such cases the preparations of this kind are invaluable.

---

**Metramine:** Palatinoids.—OPPENHEIMER, SON & CO., London. This drug is described as a highly-purified form of hexamethylene tetramine, formed by the action of ammonia on formaldehyde. It is believed to liberate formaldehyde in the body, and to this may be ascribed its value as a urinary antiseptic. The drug has received a great variety of trade names, but the palatinoid form is a very convenient one, and it should have a great field of utility in all suppurating conditions of the urinary tract. It is sufficiently powerful to destroy gonococci, as well as typhoid and other bacilli.

The compound palatinoid containing with metramine (gr. iv.) small doses of sandal oil and copaiba should be of great benefit in cases of persistent gonorrhoea.

---

**Membroids.**—EVANS SONS, LESCHER & WEBB, London.—We have received a sample of these preparations. The drug is enclosed in a thick membranous envelope, which is capable of resisting acid digestion in the stomach, whereas in the alkaline pancreatic juice it readily digests, and therefore the drug is not likely to give rise to any gastric disturbance, but will be readily absorbed from the small intestine. We have found that the membrane is very insoluble in acid fluids, but that it readily digests in pancreatic solution. This form of administration must commend itself for the treatment of intestinal conditions, and in cases where nauseous eructations are commonly the result of a liberation of the drug in the stomach.

**Holadin:** the entire pancreas gland extract.—FAIRCHILD BROS. & FOSTER, New York.—In capsules containing three grains.

This powder possesses great tryptic activity, and contains also the amylotic and lipolytic enzymes. It contains the important cell constituents, lecithin and nuclein, and is put up in a very convenient form for such cases in which pancreatic activity is deficient.

**Lotio Pancreatis:** a trypsin surgical solvent.—It is prepared from the fresh pancreas, and contains the trypsin enzyme in a stable solution. It is to be used twice daily, after dilution with an equal volume of distilled water.

---

**Glycerole Trypsin.**—ARMOUR & CO., Chicago.—This is a similar fluid, containing the pancreatic ferments in permanent solution, ready either for topical surgical use or for internal medication. As a surgical solvent it may be applied without dilution.

---

**"Soloid" Black Mercurial Lotion.**—BURROUGHS, WELLCOME & CO., London.—"Soloid" Black Mercurial Lotion presents a very convenient means of preparing a mercurial or black lotion, the value of which is well recognised in condylomata and other syphilitic manifestations, in pruritus and in many other skin affections. One, powdered and shaken with one fluid ounce of water, makes a lotion corresponding in strength of active ingredient to Lotio Hydrarg. Nigra, P.B. The official preparation may be more closely approximated by the addition of 24 minims of glycerine to the fluid ounce.

---

**Elixir Combreti.**—HAROLD E. MATTHEWS, Clifton.—Some attention has been attracted of late by a new drug "Combretum," consisting of the leaves and twigs of *Combretum Sundaicum*, introduced as a remedy for the opium habit. *Combretum Sundaicum* is a powerful climber growing abundantly in the state of Selangor, Malay Peninsula. The Chinese coolies use a decoction of the roasted leaves and twigs to counteract the craving for opium, with, it is stated, great success. A chemical investigation has been made which showed tannins and resins to be present, but no alkaloid and only a trace of a glucoside. As the glucoside is not found in the roasted drug, it is unlikely that the properties of the drug are due to it. Elixir Combreti is an elegant, non-alcoholic preparation ,of agreeable taste, containing the water-soluble constituents of the drug. It is administered in doses of two fluid drachms, the patient's allowance of opium being concurrently diminished.

---

**Collargol:** a colloidal silver preparation, Heydan-Credé.—E. W. BLASIUS, London.—This non-poisonous antiseptic is recommended for the internal treatment of septic diseases as well as for the cleansing and dressing of wounds. Tablets, containing $\frac{3}{4}$ gr. of Collargol, are of use for internal medication, and tablets containing 4 grs. are convenient for the preparation of solutions and enemata. It is also made up as Unguentum Credé for external use.

---

**Carbonic Acid Baths :** A Zucker's system.—THE HYGIENIC COMPANY, 26 Southwark Bridge Road, London.—The Nauheim system has given rise to a demand for the use of baths in which the generation of carbonic acid in the bath is an essential element. It is not always possible to go to Nauheim, but it is easy to take a carbonic acid bath at home. A bottle of the formic acid is put into the bath water, and two pads containing the alkali continue to give rise to constant and regular effervescence in the bath for a considerable time. The water becomes saturated with the gas, a portion of which is deposited in globules on the patient's skin. The temperature of the water should be about 93° Fahr. The method has the merit of being simple, easy and effective.

